# A Seven-Year-Old Girl With Dysphagia Due to Fear of Swallowing: A Favorable Outcome With Cognitive Behavioral Therapy Using an Anxiety Hierarchy Chart

**DOI:** 10.7759/cureus.33196

**Published:** 2022-12-31

**Authors:** Mika Etoh, Tomoyo Itonaga, Saori Oguri, Akio Kiyota, Kenji Ihara

**Affiliations:** 1 Department of Pediatrics, Oita University Faculty of Medicine, Yufu, JPN; 2 Department of Child Psychiatry, Oita Rehabilitation Center, Oita, JPN

**Keywords:** arfid, cognitive behavioral therapy, anxiety hierarchy chart, avoidant/restrictive food intake disorder, eating disorder

## Abstract

Avoidant/restrictive food intake disorder (ARFID) is an eating disorder characterized by avoidance and aversion to food and eating. Food restriction is not due to a body image disturbance but rather to an anxiety or phobia of food and eating or abnormal hypersensitivity to food, such as its texture, taste, or smell, or a lack of interest in food/eating. We herein report a seven-year-old girl with dysphagia due to a fear of swallowing with a favorable outcome thanks to cognitive behavioral therapy using an anxiety hierarchy chart. After a scary experience of seeing her bother choking on a sausage, the patient struggled with a strong fear of eating, especially swallowing, and was diagnosed with ARFID. We constructed a hierarchical chart of food insecurity, listing her favorite sweets in order, from soft to hard. She picked out daily sweets and snacks from the list. She gradually learned to eat hard-shaped food, achieved an adequate oral calorie intake, and was discharged on the twenty-second hospital day. This case indicates that cognitive behavioral therapy using the anxiety hierarchy chart can be applied to the treatment of school-age children with ARFID.

## Introduction

Eating disorders are a heterogeneous group of complex mental disorders characterized by unusual eating behavior. The major disorders listed in the "Feeding and Eating Disorders" group of the Diagnostic and Statistical Manual of Mental Disorders-5 (DSM-5) include anorexia nervosa and bulimia nervosa, and these disorders account for about half of all eating disorders in children [1,2]. Avoidant/restrictive food intake disorder (ARFID), previously referred to as "Eating disorder not otherwise specified," is a relatively uncommon type among eating disorders [3] and is characterized by food avoidance due to indifference to food, choking, anxiety about dysphagia and vomiting, and a narrow range of food preferences. It is not characterized by body image disturbance [2]. ARFID is classified as “other specified feeding and eating disorder” in the guideline of eating disorders [4].

Cognitive behavioral therapy (CBT) has been widely applied as a therapeutic modality in general psychiatric practice. This is defined as psychotherapy that combines cognitive and behavioral therapy by identifying defective or maladaptive patterns of thinking, emotional responses, or behavior and replacing them with desirable patterns of thinking, emotional responses, or behavior. Exposure therapy is categorized as CBT and is most commonly applied to treat obsessive-compulsive disorder, posttraumatic stress disorder (PTSD), and phobias. It has also been reported to be effective for ARFID [5-7]. Since exposure therapy often uses hierarchical anxiety charts, it seems reasonable to use them in the CBT for ARFID.

We herein report a seven-year-old girl with ARFID, for whom a hierarchical anxiety chart based on food preference was effectively applied in the treatment of food phobia.

## Case presentation

The patient had been born at 40 weeks of gestational age. She had normal growth and development during her infancy. She was diagnosed with Tourette's disorder at five years of age. About half a year later, she developed motor and phonic tics, and behavioral intervention with play therapy was started. Afterward, the tics gradually decreased, and had almost disappeared by six years and 10 months old, so the intervention was finished. No problematic behavior or learning attitude had been pointed out in kindergarten or school. At around the same time, as she was diagnosed with Tourette’s disorder, her younger brother was also diagnosed with autism spectrum disorder at three years of age. With the exception of the patient’s brother, there was no family history of neurodevelopmental disorder.

At seven years and one month old, she was shocked to see her brother choking on a sausage. Thereafter, her food intake gradually decreased, as she suffered from a feeling of a foreign object being stuck in her throat with discomfort in her chest. She could not eat anything, only lick candy and drink a little water. Nine days after the event, the patient consulted a local clinic and underwent intravenous fluid therapy at an outpatient ward. The patient still took little food and lost 1 kg of weight in five days, and on Day 14 after the event, she was referred to our hospital.

On admission, she was 127.0 cm (+1.7 SD) in height and 24.7 kg (+0.7 SD) in body weight. She showed slight weight loss, with an ideal weight of 94% without findings of dehydration. She smiled during conversations with the medical staff. A blood test showed no electrolyte imbalances or dehydration (Table 1). Head-to-neck magnetic resonance imaging showed no abnormalities. Her full-scale intelligence quotient (IQ) was 98, without marked variation in subscales (verbal comprehension index of 105, perceptual reasoning index of 100, working memory index of 85, and processing speed index of 99) according to the fourth edition of the Wechsler Intelligence Scale for Children (WISC IV) test. The Autism-Spectrum Quotient score was 15.4 (subscale scores: social skill index, 2; attention switching index, 2; local details index, 4; communication index, 4.4; and imagination index, 3), which was within the normal range (cut-off score 20).

**Table 1:** Blood data of laboratory examinations at admission AST, aspartate aminotransferase; ALT, alanine aminotransferase; BUN, blood urea nitrogen; CRP, C-reactive protein; fT4, free thyroxine; fT3, free triiodothyronine; LH, luteinizing hormone; FSH, follicle stimulating hormone; IGF-1, insulin-like growth factor 1; WBC, white blood cell count; RBC, red blood cell count

Test	Result	Unit	Reference Values
【Blood count】			
WBC	6,970	/µL	5,000-14,500
RBC	458×10^4	/µL	450×10^4-510×10^4
Hemoglobin	13.4	g/dl	12-14
Platele	25×10^4	/µL	15×10^4-40×10^4
【Biochemistry】			
Total protein	7.3	g/dL	6.3-8.0
Albumin	4.6	g/dL	3.7-5.7
AST	28	IU/L	16-38
ALT	12	IU/L	4-25
Toal-cholesterol	166.3	mg/dL	<190
BUN	8.6	mg/dL	6-20
Creatinine	0.45	mg/dL	0.28-0.49
Glucose	103	mg/dL	70-109
CRP	0.01	mg/dL	<0.3
Sodium	138	mEq/L	138-145
Potassium	4.1	mEq/L	3.4-4.7
Chloride	105.3	mEq/L	98-106
Calsium	9.46	mg/dL	9.4-10.3
Inorganic phosphorus	4.43	mg/dL	3.6 - 5.8
Copper	75.3	μg/dL	78.2 - 118.8
Magnecium	2.4	mg/dL	1.8 - 2.2
Zinc	83.2	μg/dL	80 - 130
【Endocrine】			
Thyrotropin	1.68	µIU/mL	0.32 - 4.0
fT4	1.34	ng/mL	1.04 - 2.01
fT3	2.91	pg/dL	2.49 - 4.54
LH	<0.3	mIU/mL	0.1 - 1.5
FSH	2.94	mIU/mL	0.5 - 7.5
IGF-1	110	ng/mL	89 - 357

**Figure 1:**
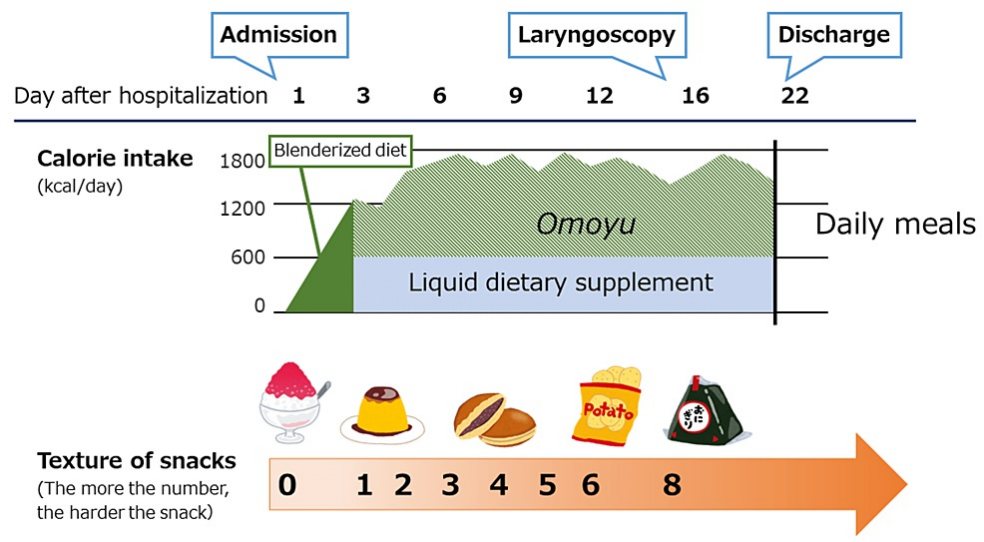
Clinical chart of the patient *Omoyu* is a Japanese traditional liquid food; it is the supernatant of a thin rice porridge cooked with a large amount of water.

At admission, however, she claimed that she did not want to eat any food, as she was scared of swallowing it, and instead requested intravenous nutrition, as she did not want to lose any more weight. We started to treat her with a blenderized diet to maintain her calorie intake per os. However, she only ate a little food under a strong fear of swallowing. On the third day of hospitalization, all daily meals were changed to *omoyu* (Japanese traditional liquid food; the supernatant of a thin rice porridge cooked with a large amount of water) with liquid dietary supplements. She was then able to take a liquid-type diet orally at approximately 1,600 kcal per day (Figure 1).

On the fourth day, we made a hierarchical chart of anxiety about food based on an interview with her; her favorite sweets and snack were listed from soft to hard type in an orderly manner and displayed at her bedside (Table 2). We let her pick out her sweets from the list at every snack time, gradually increasing from soft to harder types. On Day 16, laryngoscopy detected no abnormal findings, which also helped decrease her anxiety. She successfully ate a rice ball on the same day. On Day 22 of hospitalization, she had sufficient oral intake and was discharged from the hospital. Follow-up interviews were conducted at the outpatient clinic to confirm that she was obtaining sufficient calories from daily meals at home.

**Table 2:** Anxiety hierarchy chart for the patient

No.	Name of snacks	Texture of snacks
0	cocoa spread, shaved ice	Soft
1	pudding	↓
2	chocolate chip ice cream	↓
3	castella, waffle	↓
4	gummies, sweet potato cake	↓
5	Dorayaki (bean-jam pancake), French fries	↓
6	potato chips	↓
7	candied sweet potato	↓
8	rice ball	↓
9	chewy bread	↓
10	skewer dumplings, mochi (rice cake) ice cream	Hard

## Discussion

ARFID is a new diagnosis in DSM-5, being previously referred to as "Eating disorder not otherwise specified." ARFID is similar to anorexia nervosa in that both disorders involve restricting the amount and types of food one consumes, but unlike anorexia, ARFID does not involve any distress with regard to body shape or size or fears of becoming fat [1]. Clinical features of ARFID include a history of vomiting and fear of choking, hypersensitivity, and low interest in food. In addition, a high rate of comorbid anxiety disorders (58.3%-72%) and neurodevelopmental disorders (27%) have been reported [8-10]. No consensus exists on the appropriate length of hospitalization for pediatric patients with ARFID or other eating disorders. Several studies have reported that patients with ARFID experience a longer duration of illness and often require more enteral nutrition in comparison to patients AN [9-11], whereas other studies reported that there was no difference in the length of hospitalization between patients with ARFID and those with AN [12,13]. In the two previous reports, the mean length of hospital stay of ARFID patients was 73.9 ± 69.9 and 182 ± 102 days, respectively.

The present patient had a strong fear of eating food, resulting in an insufficient caloric intake. At the beginning of hospitalization, we attempted reconstruction of the cognitive function concerning food through an interview but failed. We then performed CBT based on previous reports that CBT could be used to treat anxiety disorders and phobias in children over seven years old [5-7,14-17]. Among various CBT techniques, such as psychoeducation and cognitive restructuring, we decided to apply exposure therapy to our patient.

Exposure therapy corrects pathologic thoughts by exposing patients to the causes of anxiety. To recover from abnormal thoughts, patients must detect discrepancies between their expectations (what might happen) and the outcomes (what actually happens) by themselves. It is important to determine the patient's specific expectations before the onset of exposure and to record their confidence in those expectations. This technique can help reduce self-destructive thinking. For the present case, the patient remained interested in eating sweets, so we speculated that exposure therapy to sweets would be easy for her to overcome. An anxiety hierarchy chart based on the hardness of sweets and similar foods was then created, and stepwise exposure therapy was carried out using the chart. By gradually increasing the firmness of these foods and repeatedly reassuring her that nothing had gone wrong, she was able to switch from soft to hard sweets and food.

We considered this strategy to have two key points; first, the patient herself was involved in creating the chart and choosing daily sweets and foods, thus allowing her to develop a positive attitude toward dietary treatment; second, the patient was able to visualize her own progress in treatment by looking at the hierarchy chart of anxiety at her bedside. Given these findings, we concluded that CBT using this chart was effective in treating this patient. Her length of hospitalization (22 days) seemed relatively short in comparison to previous reports of pediatric ARFID patients [12,13]. We, therefore, conclude that the use of the anxiety hierarchy chart might help reduce hospital stays, however, it is not easy to compare our case and other cases in previous reports because the indications for hospitalization may have been highly variable among cases.

Additional information on this case should be noted. For example, no significant episodes suggestive of neurodevelopmental disorders were observed during the CBT in the hospital, but a history of Tourette's disorder, a family history of autism spectrum disorder, and poor working memory suggested an underlying neurodevelopmental disorder. Although our patient was not evaluated by the pediatric psychiatrist during hospitalization, her anxiety temperament may have existed in the psychological background of her phobia of swallowing. We need to continue developmental support, as she might manifest a developmental disorder in the future.

## Conclusions

We applied exposure therapy to our patient using the anxiety hierarchy chart with dysphagia due to fear of swallowing. CBT using an anxiety hierarchy chart might be an optional approach for managing child-age ARFID.
